# Intraoperative Evaluation of Breast Tissues During Breast Cancer Operations Using the MasSpec Pen

**DOI:** 10.1001/jamanetworkopen.2024.2684

**Published:** 2024-03-22

**Authors:** Kyana Y. Garza, Mary E. King, Chandandeep Nagi, Rachel J. DeHoog, Jialing Zhang, Marta Sans, Anna Krieger, Clara L. Feider, Alena V. Bensussan, Michael F. Keating, John Q. Lin, Min Woo Sun, Robert Tibshirani, Christopher Pirko, Kirtan A. Brahmbhatt, Ahmed R. Al-Fartosi, Alastair M. Thompson, Elizabeth Bonefas, James Suliburk, Stacey A. Carter, Livia S. Eberlin

**Affiliations:** 1 Department of Chemistry, The University of Texas at Austin; 2 Michael E. DeBakey Department of Surgery, Baylor College of Medicine, Houston, Texas; 3 Department of Pathology and Immunology, Baylor College of Medicine, Houston, Texas; 4 Department of Biomedical Data Science, Stanford University, Stanford, California

## Abstract

**Importance:**

Surgery with complete tumor resection remains the main treatment option for patients with breast cancer. Yet, current technologies are limited in providing accurate assessment of breast tissue in vivo, warranting development of new technologies for surgical guidance.

**Objective:**

To evaluate the performance of the MasSpec Pen for accurate intraoperative assessment of breast tissues and surgical margins based on metabolic and lipid information.

**Design, Setting, and Participants:**

In this diagnostic study conducted between February 23, 2017, and August 19, 2021, the mass spectrometry–based device was used to analyze healthy breast and invasive ductal carcinoma (IDC) banked tissue samples from adult patients undergoing breast surgery for ductal carcinomas or nonmalignant conditions. Fresh-frozen tissue samples and touch imprints were analyzed in a laboratory. Intraoperative in vivo and ex vivo breast tissue analyses were performed by surgical staff in operating rooms (ORs) within 2 different hospitals at the Texas Medical Center. Molecular data were used to build statistical classifiers.

**Main Outcomes and Measures:**

Prediction results of tissue analyses from classification models were compared with gross assessment, frozen section analysis, and/or final postoperative pathology to assess accuracy.

**Results:**

All data acquired from the 143 banked tissue samples, including 79 healthy breast and 64 IDC tissues, were included in the statistical analysis. Data presented rich molecular profiles of healthy and IDC banked tissue samples, with significant changes in relative abundances observed for several metabolic species. Statistical classifiers yielded accuracies of 95.6%, 95.5%, and 90.6% for training, validation, and independent test sets, respectively. A total of 25 participants enrolled in the clinical, intraoperative study; all were female, and the median age was 58 years (IQR, 44-66 years). Intraoperative testing of the technology was successfully performed by surgical staff during 25 breast operations. Of 273 intraoperative analyses performed during 25 surgical cases, 147 analyses from 22 cases were subjected to statistical classification. Testing of the classifiers on 147 intraoperative mass spectra yielded 95.9% agreement with postoperative pathology results.

**Conclusions and Relevance:**

The findings of this diagnostic study suggest that the mass spectrometry–based system could be clinically valuable to surgeons and patients by enabling fast molecular-based intraoperative assessment of in vivo and ex vivo breast tissue samples and surgical margins.

## Key Points

**Question:**

Are molecular profiles acquired by the MasSpec Pen predictive of disease state in healthy and cancerous breast tissue?

**Findings:**

In this diagnostic study, the MasSpec Pen was used for molecular analysis of 143 fresh-frozen healthy and invasive ductal carcinoma tissue samples in a laboratory and during in vivo and ex vivo testing in 25 surgical cases. High agreements with pathology findings were achieved for statistical prediction of healthy breast and disease state in banked, freshly excised, and in vivo tissue samples.

**Meaning:**

These findings suggest that the described technology and statistical classifiers may be useful for in vivo and ex vivo cancer detection during breast cancer operations.

## Teaser

This diagnostic study evaluates the performance of a handheld mass spectrometry device used for in vivo and ex vivo molecular analysis during breast cancer operations to discriminate healthy breast tissue from invasive ductal carcinoma.

## Introduction

Surgical resection is considered the standard of care for patients diagnosed with breast cancers.^1^ Patients with early-stage breast cancers may undergo breast conserving surgery (BCS), which involves either complete tumor resection with a rim of healthy tissue while preserving the remaining healthy breast tissue or mastectomy. In both procedures, complete tumor removal is paramount to patient outcomes, as achieving negative surgical margins offers the greatest potential for prolonged disease-free survival.^2,3,4,5^ Surgical margins of the resected breast specimen are traditionally evaluated postoperatively by microscopic histopathologic assessment of fixed tissue sections, with negative margins considered “no tumor on ink” for invasive ductal carcinoma (IDC).^3^ However, clinical studies report positive margins in approximately 20% to 40% of BCS.^3,6^ Re-excisions of the positive margin are often recommended to ensure complete cancer removal but are associated with increased risk of morbidity and patient distress, poorer aesthetic outcomes, and higher health care costs.^7,8,9,10^ Intraoperative surgical margin evaluation techniques such as frozen section analysis,^11,12^ spectroscopy,^13,14^ and imaging,^15,16^ among others,^17,18^ have been developed to help address challenges encountered during surgical margin evaluation. Yet, these techniques are not routinely used for intraoperative margin evaluation, as they require specialized training for operation, their accuracy and preference of use varies across institutions, and their implementation can be time- and labor-intensive.^17,19^

Several emerging molecular-based technologies have been proposed for intraoperative analysis of breast cancer margins.^17,18^ Mass spectrometry (MS) techniques, in particular, offer the unique opportunity to incorporate highly specific molecular information into the surgical environment to guide surgeons in tumor resection. For instance, direct MS techniques have been used to image ex vivo breast tissue sections or to analyze excised breast cancer tissue samples in the operating room (OR), enabling detection of cancer-associated metabolic alterations.^20,21,22,23^ Handheld MS-based approaches have also been developed for surgical use.^24,25^ Rapid evaporative ionization MS, for example, has been used to investigate molecular profiles of banked healthy and cancerous breast tissue samples and demonstrated intraoperative use in BCS in proof-of-concept studies.^24,26^

We have previously developed the MasSpec Pen (MSPen), a handheld biocompatible device integrated to a mass spectrometer for direct, rapid (approximately 15 seconds) molecular analysis of human tissues.^25,27,28,29,30^ The system uses a droplet of solvent to gently extract endogenous metabolites and lipids from tissues without causing tissue damage. The droplet is then transferred to and analyzed by a high-performance mass spectrometer to produce molecular data characteristic of tissue type and disease state. This technology provides surgeons with the capability for nondestructive assessment of the metabolic composition of tissues in vivo prior to resection and the excised specimen ex vivo to help identify tissues and inform surgical decision-making. The described technology has been successfully translated to the OR for clinical feasibility testing in open operations and for surgical margin evaluation in pancreatic cancer surgeries.^28,29^ Herein, we describe the application of the MSPen technology and multivariate statistical classification for the discrimination of healthy breast tissues from IDC tissues based on their metabolic profiles and further evaluate its performance on mass spectra collected intraoperatively by surgeons in vivo and ex vivo during breast operations.

## Methods

### Banked Tissue Samples

In this diagnostic study, deidentified, banked, frozen healthy breast and IDC tissue samples were retrospectively obtained from the Cooperative Human Tissue Network (CHTN) from February 23, 2017, to July 24, 2018, under a protocol approved by The University of Texas at Austin institutional review board (IRB). Banked specimens purchased from CHTN were collected under either waiver of consent or surgical consent, per CHTN policy. This study followed the Transparent Reporting of a Multivariable Prediction Model for Individual Prognosis or Diagnosis (TRIPOD) guideline.^31^

### Analysis of Banked Breast Tissue Samples

The handheld sampling device coupled to a mass spectrometer (Q Exactive; Thermo Fisher Scientific) was used to analyze the tissue samples in 4 batches in a research laboratory. For laboratory experiments, a tubing length of 1.5 m and a pen tip reservoir diameter of 2.7 mm (area, 5.73 mm^2^) was used. Experimental details are in eMethods 1 in Supplement 1. Following analysis, a surgical ink stain was applied to the tissue to delineate the precise region analyzed for a direct correlation of histology with the mass spectra obtained. Samples were immediately flash frozen, sectioned (5-10 μm), and stained with hematoxylin and eosin (H&E) for blind pathologic evaluation by a breast pathologist.

### Intraoperative Clinical Testing of the MSPen During Breast Cancer Operations

The diagnostic study was conducted at Baylor St Luke’s Medical Center and O’Quinn Medical Tower (Houston, Texas) from October 2, 2018, to November 20, 2019, and May 11 to August 19, 2021, respectively, under approved IRB protocol from the Baylor College of Medicine (BCM) and The University of Texas at Austin. Written informed consent was obtained (eMethods 2 in Supplement 1). Adult patients scheduled to undergo breast surgery for IDC, ductal carcinoma in situ (DCIS), or nonmalignant conditions were eligible for enrollment. Race and ethnicity data were collected for this study via electronic health record. Race and ethnicity were not taken into consideration during the statistical analyses and were solely collected for grant reporting purposes. Race categories were Asian, Black, Native Hawaiian or Other Pacific Islander, and White, and ethnicity categories were Hispanic or Latino and not Hispanic or Latino. Limited sample size in this pilot study precluded any analysis by race and ethnicity. Eligibility criteria are further described in eMethods 2 in Supplement 1. Technical details of the clinical study can be found in a previous publication.^29^

During surgery, autoclaved sampling devices were placed in the surgical field for in vivo tissue analyses and next to the mass spectrometer for ex vivo analyses of freshly excised specimens. The attending surgeons (A.M.T., E.B., S.A.C.) performed in vivo analyses of tissue regions of interest based on the surgeon’s gross assessment, including surgical margins of the suspected tumor, healthy breast tissue, and surgical cavity following tissue resection. When possible, in vivo and ex vivo analyses of lymph nodes were also performed. Analyses were then compared with the postpathology reports for those regions. No ink was used in the intraoperative analyses. Statistical classification was not performed on intraoperative analyses obtained from lymph node tissue. Research personnel from BCM (C.P., K.A.B., A.R.A.-F.) performed ex vivo analyses of the surgical margins of the freshly excised specimens. Data were not communicated to the surgical staff. No statistical predictions were performed during the operations.

### Analysis of Prospectively Collected Touch Imprints

Touch imprints were collected from patients with confirmed IDC, or with no residual carcinoma as controls, who provided written consent under the protocol approved by the Baylor College of Medicine IRB as an a priori, exploratory, prospective pilot study. Samples were prepared by a pathologist (C.N.) by pressing the breast tissue specimen against a histologic slide. Slides were frozen at −80 °C and thawed prior to analysis. Samples were analyzed using the handheld sampling device coupled to a Q Exactive HF mass spectrometer (Thermo Fisher Scientific) in a research laboratory at BCM using the same experimental parameters previously described. Following analysis, samples were H&E stained and evaluated by a pathologist (C.N.).

### Statistical Analysis

**Figure 1.**
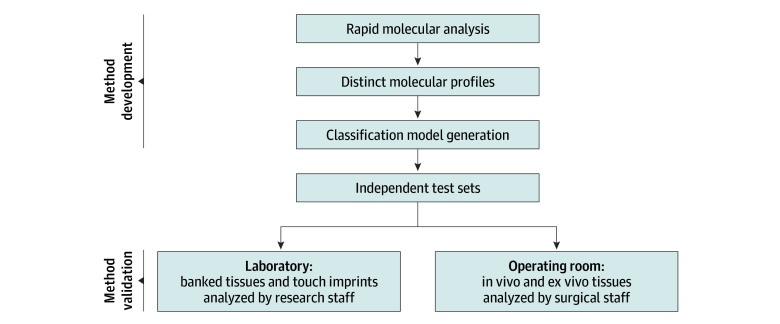
Development of the MasSpec Pen and Classification Model for Tissue Analysis and Surgical Margin Evaluation in Breast Cancer Operations

Preprocessing and statistical analysis of banked and intraoperative data have been previously described, with additional details described in eMethods 3 in Supplement 1.^28,32^ In brief, logistic regression regularized using the least absolute shrinkage and selection operator (lasso) was implemented via the glmnet package (version 2.0-16) in the R CRAN language library (R Project for Statistical Computing) to develop a classification model using only banked tissue samples. Samples were labeled by the pathologist as healthy or IDC based on the specific histology within the MSPen sampling region. The mass spectra collected from the tissue samples in the first 2 batches of experiments were randomly split into a training and a validation set. The lasso model was generated using the training set of data via leave-one-out cross-validation and used for all predictions for the remainder of this study. Accuracy, sensitivity, and specificity of prediction results were calculated based on agreement with pathologic evaluation of the same tissue samples. We then evaluated the performance of the classification model generated from banked tissue specimens on mass spectra collected intraoperatively and mass spectra collected from touch imprints as fully independent test sets (Figure 1). Student *t* tests with Benjamini-Hochberg correction were performed, with 2-sided *P* < .05 considered significant.

## Results

### Molecular Analysis and Statistical Prediction of Healthy Tissue and IDC in Breast Tissue Samples

**Figure 2.**
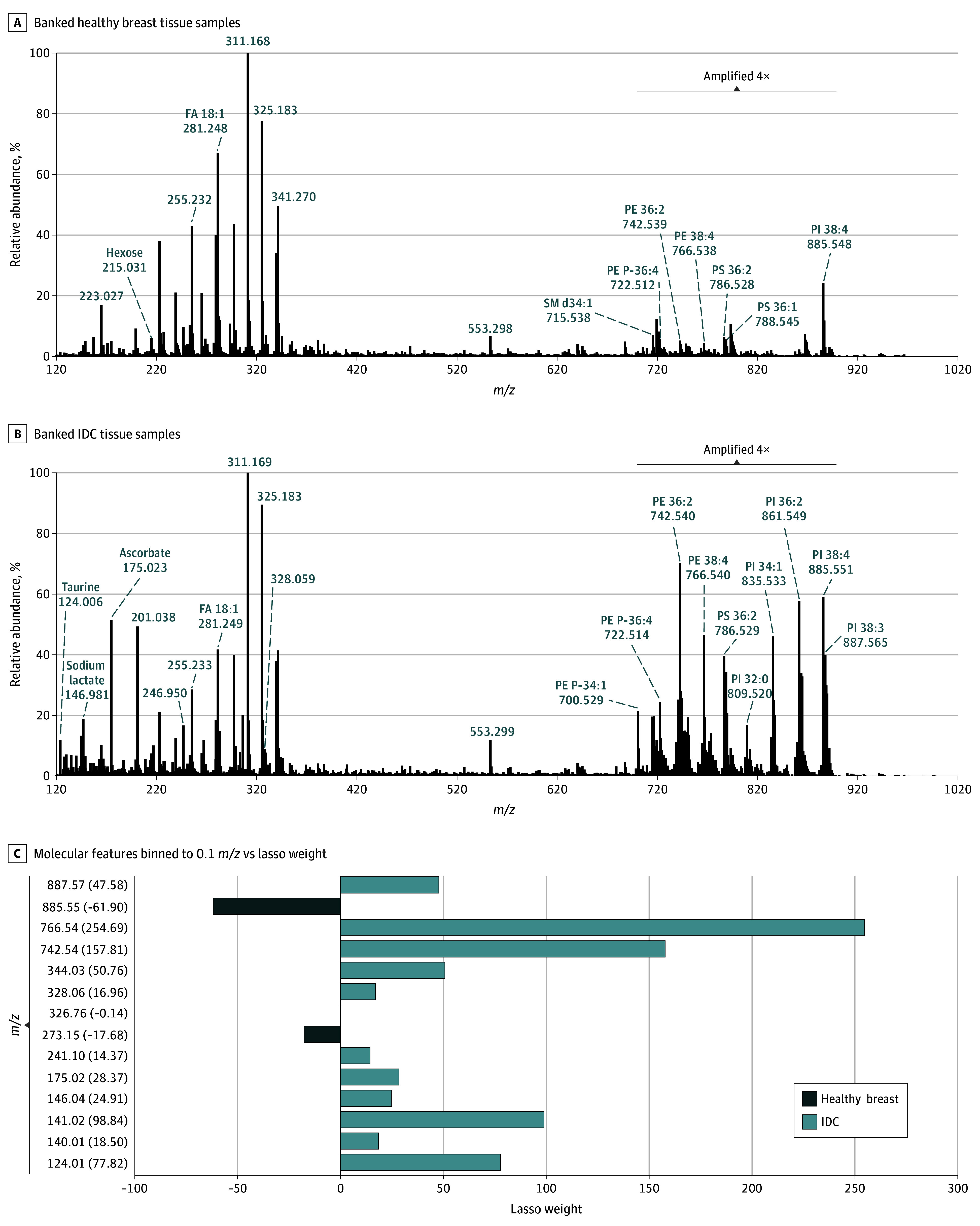
Representative Metabolite and Lipid Profiles Collected From Banked Breast Tissue Samples and Classification Model Performance for Prediction of Invasive Ductal Carcinoma (IDC) Using Banked Tissue Samples A and B, Data from healthy breast (n = 43) and IDC (n = 25) tissue samples were averaged to generate a representative mass spectrum. The lipid region (*m*/*z* 700-900) was amplified 4 times in both mass spectra to enhance data visualization. C, Each molecular feature is presented as *m*/*z*, with its corresponding lasso weight in parentheses. FA indicates fatty acid; SM, sphingomyelin; PE, phosphatidylethanolamine; PI, phosphatidylinositol; and PS, phosphatidylserine.

Analysis of the 143 banked tissue samples (79 healthy breast and 64 IDC), analyzed in 4 total batches, yielded rich molecular profiles composed of a diverse array of metabolites and complex glycerophospholipid (GP) species (Figure 2A and B). For example, small metabolites including ascorbic acid (*m*/*z* 175.023), hexose (*m*/*z* 215.031), glutathione (*m*/*z* 306.076), and glutamate (*m*/*z* 146.046) and fatty acids (FAs) including FA 16:0 (*m*/*z* 255.232) and FA 18:1 (*m*/*z* 281.249) were detected in both tissue types. Lipids from various GP classes were detected, including phosphatidylinositol (PI), phosphatidylethanolamine (PE), and phosphatidylserine (PS). Qualitatively, the mass spectra obtained from IDC tissue samples showed a higher relative abundance of GP species in general compared with healthy breast tissue samples, particularly PI species such as PI 34:1 (*m*/*z* 835.533), PI 36:1 (*m*/*z* 861.549), and PI 38:3 (*m*/*z* 887.565). The changes in relative abundance of several lipids and metabolites between the healthy and IDC tissue samples were statistically significant, as shown for selected species in eFigure 1 in Supplement 1.

Healthy breast tissue was composed of adipose, connective tissue, and luminal cells lining healthy breast ducts. The major histological component of IDC tissues was tumor cells, although lymphocytes, DCIS, and stroma were frequently present as well. Using the lasso method to generate a statistical model from the data collected, 14 molecular species were selected as predictive ions to classify healthy breast tissue and IDC (Figure 2C), including PI 38:4 (*m*/*z* 885.549) and 2 unidentified molecules (*m*/*z* 273.146 and *m*/*z* 326.760) weighted toward healthy breast tissue and ascorbic acid (*m*/*z* 175.023), PE 36:2 (*m*/*z* 742.539), and PE 38:4 (*m*/*z* 766.540) weighted toward IDC.

The model exhibited high predictive performance on the training set (43 healthy breast tissue samples, 25 IDC samples), yielding 100% sensitivity (0% false-negative rate [FNR]), 93.0% specificity (7.0% false-positive rate [FPR]), and 95.6% overall agreement with pathology. On the validation set (14 healthy breast tissue samples, 8 IDC samples), 100% sensitivity (0% FNR), 92.9% specificity (7.1% FPR), and 95.5% accuracy were achieved, with all but 1 sample (4.5%) predicted correctly compared with pathology. We further evaluated the predictive performance of the classification model by using an independent test set of 53 samples from batches 3 and 4, achieving a sensitivity of 83.9% (16.1% FNR), a specificity of 100% (0% FPR), and accuracy of 90.6%, with 5 IDC samples (9.4%) misclassified as healthy breast tissue. Full prediction results are summarized in eTable 1 in Supplement 1.

### Intraoperative Analysis During Breast Operations

**Figure 3.**
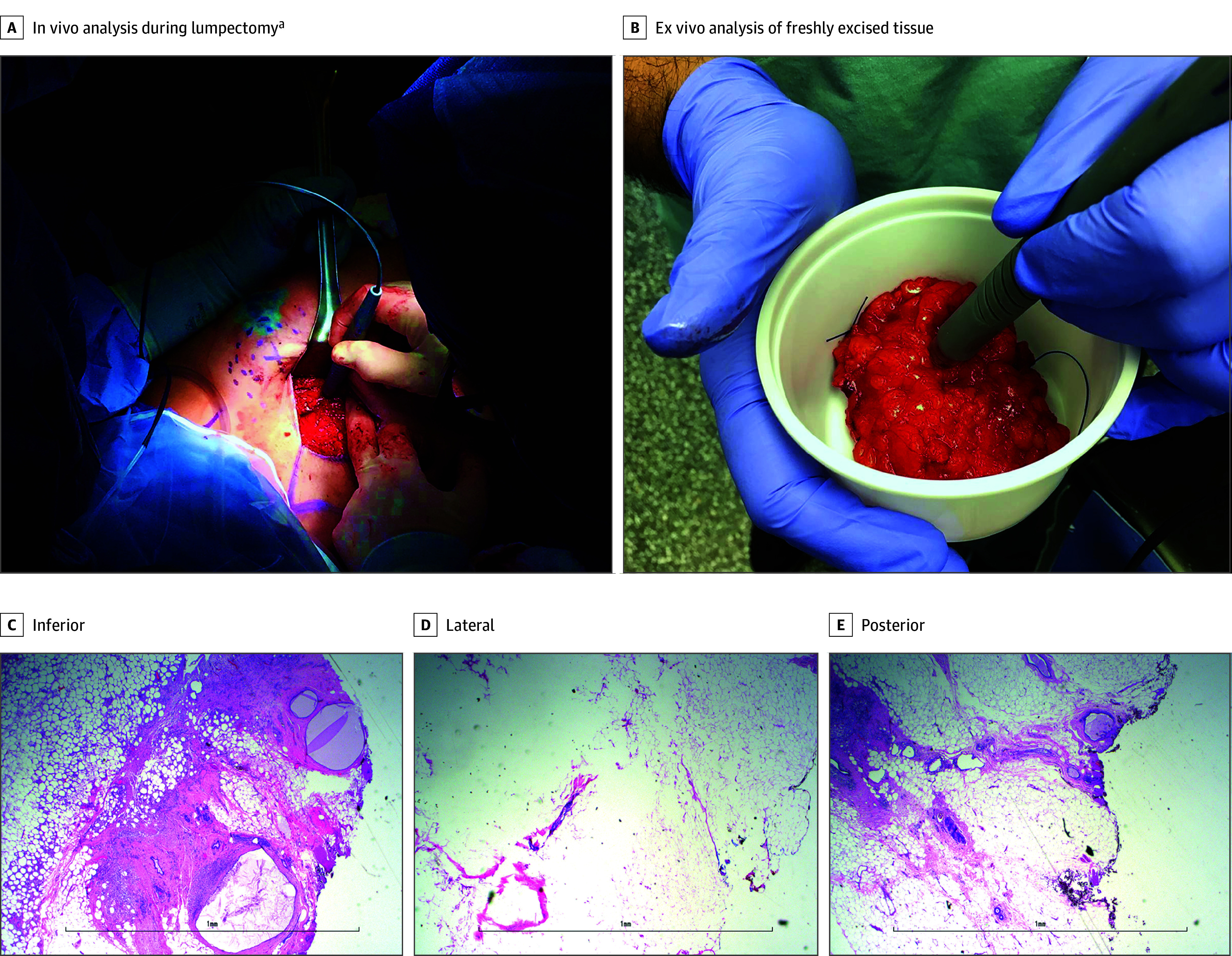
Intraoperative Tissue Analysis With the MasSpec Pen During Breast Operations C-E, Optical images of hematoxylin and eosin–stained, formalin-fixed, paraffin-embedded tissue sections of surgical margins analyzed with the MasSpec Pen during a re-excision surgery (case Br0030). Scale bar is 1 mm. ^a^Image reused with permission from Zhang et al.^29^

**Figure 4.**
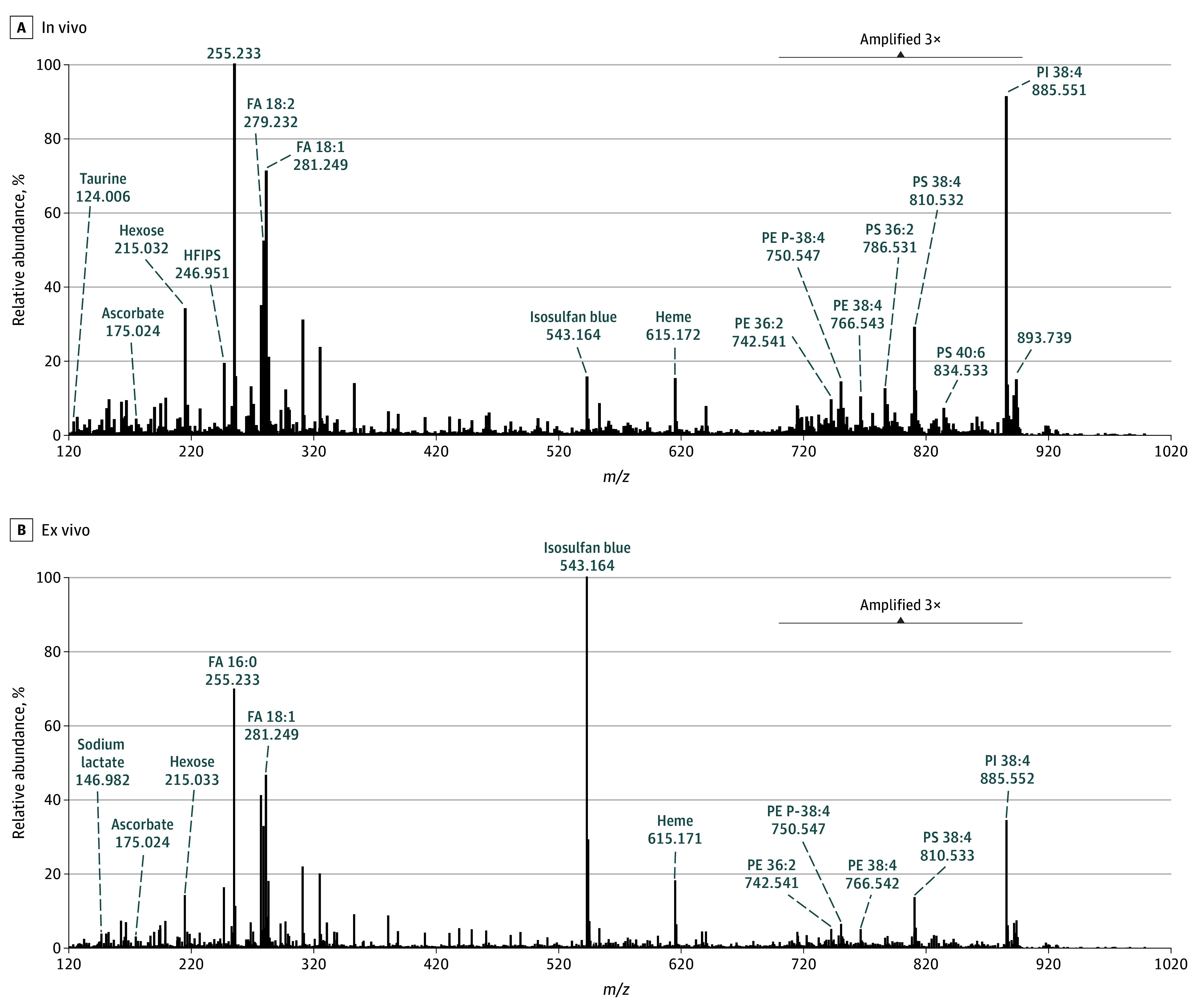
Molecular Profiles Collected Intraoperatively During Human Breast Operations Mass spectra from in vivo (n = 66) and ex vivo (n = 81) analyses, respectively, were averaged to generate representative profiles. The lipid region (*m*/*z* 700-900) was amplified 3 times in both mass spectra to enhance data visualization. FA indicates fatty acid; PE, phosphatidylethanolamine; PI, phosphatidylinositol; and PS, phosphatidylserine.

The handheld MS-based device was then translated to the OR and used by the surgeons to analyze regions of breast tissues in vivo (Figure 3A) and on freshly excised specimens (Figure 3B) during 25 breast operations—12 BCSs, 12 mastectomy operations, and 1 excisional biopsy—for patients with IDC, DCIS, and nonmalignant conditions. Patient demographics are summarized in the Table. All 25 patients were female; 1 (4%) was Asian, 4 (16%) were Black, 1 (4%) was Native Hawaiian or Other Pacific Islander, and 19 (76%) were White; 5 (20%) were Hispanic or Latino and 20 (80%) were not Hispanic or Latino. The median age was 58 years (IQR, 44-66 years). Notably, no tissue damage due to use of the system in vivo or ex vivo was noted by the surgeons or the pathology team. The intraoperative molecular profiles obtained (Figure 4) displayed metabolites, fatty acids, and GP species, such as hexose (*m*/*z* 215.032), ascorbic acid (*m*/*z* 175.023), FA 18:1 (*m*/*z* 281.249), PI 38:4 (*m*/*z* 885.551), and PS 38:4 (*m*/*z* 810.532), as was typically detected in the laboratory (Figure 2). Higher spectral noise and a higher relative abundance of blood-related species such as heme (*m*/*z* 615.172) and of isosulfan blue (Lymphazurin) (*m*/*z* 543.164), the dye used for lymph node mapping, were also detected in mass spectra obtained from intraoperative analysis of breast tissue. Similar molecular species were detected in lymph node tissue analyzed intraoperatively (eFigure 2 in Supplement 1). Statistical analysis was not performed on the data obtained from in vivo and ex vivo lymph node analyses. The time per analysis, measured from the press of the foot pedal to the end of data collection, was 37 seconds. The total analysis time per surgery depends on many factors related to the surgical case (eg, whether analyses are performed on the excised specimen and/or in the surgical cavity, the size of the sample, the total number of analyses performed, and the number of users). For example, 9 analyses were performed during surgery Br0001, resulting in a total analysis time of 5.5 minutes.

**Table.** Summary of Patient Demographic Information for All Intraoperative Cases and Prospectively Collected Touch Imprints

Characteristic	Patients^a^	
	Intraoperative cases (n = 25)	Touch imprints (n = 10)
MasSpec Pen analyses, No.	147	12
Patient age, median (IQR), y	58 (44-66)	58 (52-65)
Sex		
Female	25 (100)	10 (100)
Male	0	0
Race		
Asian	1 (4)	1 (10)
Black	4 (16)	4 (40)
Native Hawaiian or Other Pacific Islander	1 (4)	0
White	19 (76)	5 (50)
Ethnicity		
Hispanic or Latino	5 (20)	3 (30)
Not Hispanic or Latino	20 (80)	7 (70)

^a^ Data are presented as the number (percentage) of patients unless otherwise indicated.

A total of 273 intraoperative analyses were performed in the OR during 25 surgical cases, of which 126 were excluded prior to statistical analysis as described in eMethods 3 in Supplement 1. Thus, of 273 intraoperative analyses during 25 surgical cases, 147 remaining analyses from 22 cases were retained for statistical prediction. The intraoperative data obtained included 66 in vivo and 81 ex vivo analyses from different tissue regions and surgical margins. For example, for patient Br0015, who underwent a lumpectomy to treat IDC, 10 total analyses were performed, including 3 in vivo analyses of healthy breast tissue and 7 analyses of the excised specimen at the posterior margin, anterior-lateral margin, and superior margin. All analyses from patient Br0015 were predicted as healthy breast tissue by the classifier, in agreement with the postoperative pathology evaluation that reported all margins negative for carcinoma. For patient Br0001, who underwent a left mastectomy for IDC, 2 in vivo analyses (1 of healthy breast tissue and 1 of a suspected tumor) and 7 ex vivo analyses (2 of healthy breast tissue and 5 of suspected tumors) were performed. All analyses were predicted as healthy breast tissue, which agreed with the postoperative pathology evaluation that no residual carcinoma was observed within the tissue, as the patient had a complete response to neoadjuvant therapy. For another patient, Br0030, who had a re-excision procedure following positive margins after BCS, 4 in vivo analyses (2 of healthy breast tissue and 2 of inferior margins) and 6 ex vivo analyses at the lateral, inferior, and posterior margins were performed. Similar results were achieved, with all samples correctly classified as healthy tissue in agreement with postoperative pathologic reports, indicating that the re-excision BCS was successful (Figure 3C-E).

Overall, the prediction results from the intraoperative data yielded 95.9% agreement with the final pathology diagnosis, with 141 of 147 predictions in agreement with final pathology reports. Although all surgical margins were negative for carcinoma by pathology, 6 analyses of healthy breast tissue (4.1%) were classified as IDC (eTable 2 in Supplement 1). Postoperative pathology revealed that all margins evaluated during the study were negative for carcinoma. As no positive margins were accessible during the surgery and thus were not sampled, the sensitivity of the statistical classifier could not be determined for intraoperative data. Thus, we designed an exploratory pilot study using touch imprints of palpable IDC tumors (n = 9) and healthy tissues (n = 3) from patients to test the model for IDC detection. A total of 18 touch imprint analyses were collected. Of these, 12 analyses (67%) were subjected to statistical classification. Data were collected from 10 patients, all of whom were female; 1 (10%) was Asian, 4 (40%) were Black, none were Native Hawaiian or Other Pacific Islander, and 5 (50%) were White; 3 (30%) were Hispanic or Latina and 7 (70%) were not Hispanic or Latina. The median age was 58 years (IQR, 52-65 years) (Table). Although a higher background signal and lower lipid signal were observed in the molecular profiles of touch imprints (eFigure 3 in Supplement 1) compared with tissue data (Figure 2A-B and Figure 4), generally similar molecular trends were seen. Pathologic evaluation of the touch imprints identified varied sample cellularity and cell composition (eTable 4 in Supplement 1). When testing the classifier generated from banked tissue analyses to predict on this data set, 75.0% overall accuracy, 66.7% sensitivity (33.3% FNR), and 100% specificity (0% FPR) were achieved, with 9 of 12 samples classified in agreement with the pathology diagnosis (eFigure 3 and eTable 3 in Supplement 1). Two of the 3 IDC samples misclassified as healthy (66%) presented with less than 5% tumor cells and low overall cellularity in the imprint region analyzed. Sample Br0059_3, which presented 10% to 15% tumor cells, was correctly classified as IDC, as were 5 other IDC touch imprints with higher tumor cell density.

## Discussion

Accurate tissue identification and surgical margin evaluation are critical to successful breast cancer surgery and prevention of cancer recurrence.^2,4^ In this study, we evaluated the ability of a handheld mass spectrometry–based device to differentiate IDC from healthy breast tissue by generating statistical classification models for IDC detection and assessed the performance of the system intraoperatively during breast cancer operations.

The predictive model generated using data collected from tissues analyzed in the laboratory yielded 95.6% accuracy for discriminating healthy breast and IDC tissues, which is comparable with training set accuracies described in studies of tissue sections by MS imaging (approximately 98%)^21,22^ and frozen and fresh tissues by rapid evaporative ionization MS (approximately 92%-94%).^24,26^ In the validation set, our model achieved 95.5% accuracy, with all but 1 sample predicted correctly compared with pathology. Notably, 100% sensitivity was achieved, even though all IDC samples presented heterogeneous histology composed of tumor cells intermixed with collagen, adipose tissue, and necrosis within the analyzed region. The clinical performance of the IDC model was further assessed by predicting on an independent test set analyzed months after the training and validation set data were collected, resulting in overall accuracy of 90.6%. Collectively, the high clinical performance achieved by the lasso model across different sample sets analyzed with the this system suggest its utility and robustness for breast tissue classification.

The classification model consisted of various lipids and metabolites, several of which have been proposed as potential markers of breast cancer in other studies^22,33,34^ and reported to play key roles in breast cancer cell metabolism.^34,35^ For example, PI 38:4, selected by lasso as a predictive feature of healthy breast tissue, was detected at higher relative abundances in healthy breast tissues using MS imaging, whereas PI 38:3, weighted toward IDC tissue in our model, also presented in higher relative abundances in IDC tissues in the same studies.^22,33^ Another MS imaging study of breast cancer tissue sections determined that PI 38:3 was associated with breast cancer disease progression and cellular invasion.^34^ Additionally, 2 PE species, PE 36:2 and PE 38:4, that were selected as indicative of IDC tissue have been detected at higher absolute abundances in breast cancer tissue relative to healthy breast tissue using liquid chromatography MS.^35^ Polyunsaturated PE species have been previously described at increased relative amounts in breast cancer tissue compared with healthy breast tissue, corroborating our findings.^36^

We implemented this system in the OR to evaluate the clinical implications and performance of the technology for its intended use in surgical procedures. The device was incorporated into the surgical workflow and successfully used by several surgeons and clinicians with minimal training in 25 breast operations. Feedback from the surgical staff indicated the device was easy to use within the operative field and intraoperative setting. We do not anticipate that use of the device during surgery will substantially increase operation time, especially considering that this technology may provide actionable information in less time than currently available intraoperative tissue methods such as frozen section analysis.^17,19^ Notably, rich molecular data were obtained in vivo and ex vivo with no visible damage to tissue or interference to postoperative pathologic evaluation of tissue sections (Figure 3C-E). Furthermore, predictive metabolites and lipids were detected from tissue in vivo and ex vivo, despite the presence of additional biological fluids and exogenous molecules, demonstrating the robustness and clinical utility of the molecular markers for use in a clinical setting. The classifier generated from banked tissue samples was used to predict the disease state of 147 intraoperative analyses with 95.9% overall agreement vs final histopathologic reports, which is notably high for an independent test set. Overall, these results have important clinical implications, as they highlight the versatility and benefit of this technology for direct, fast margin assessment. Furthermore, these results highlight the value of molecular-based in vivo assessment of breast tissue prior to tissue excision to guide decision-making, a unique capability provided by this system that is currently unavailable to surgeons via other methods.

### Limitations

This study has limitations. Of the 147 intraoperative analyses for which disease state was predicted, 6 analyses of healthy breast tissue were classified as IDC, although the surgical margins were assessed as negative for carcinoma by pathology. The classification model generated from banked tissue samples had 93.0% specificity, meaning that false-positive results were possible for a small percentage of the analyses. We speculate that user error, instrument performance, and inherent molecular differences between fresh and frozen tissue may have been factors in these misclassifications. Inclusion of molecular data from a larger number of samples to refine the statistical classifiers may also improve predictive accuracy. Additionally, incorporation of intraoperative data into the training set has shown potential for improving the clinical performance of the model, as demonstrated previously,^28^ and will be explored in future studies following the collection of additional data, including intraoperative data from positive margins. Quality control measures are also currently being evaluated to improve method robustness and reliability, as some authors of this study have recently reported in endocrine applications.^37^

Additionally, the classification model was built using IDC samples only and thus needs to be expanded to include other histologic subtypes including DCIS, invasive lobular carcinoma, and samples of varied cellularity. Furthermore, although intraoperative analysis of a few lymph node tissue samples was performed (eFigure 2 in Supplement 1), the classification model did not include lymph nodes, and thus, statistical analysis was not pursued. Additional experiments are needed to evaluate the ability of the system to detect breast cancer metastasis in lymph node tissue, especially in challenging samples with low cellularity regions and micrometastasis.

This prospective clinical study was performed at 2 sites with a limited number of patients. Notably, no strong qualitative differences were observed between data collected at the 2 hospital sites, as the same instrument, materials, and workflows were used, as shown in eFigure 4 in Supplement 1. Larger patient cohorts and multisite studies are needed to guide diagnostic validation with appropriate statistical power and thus more rigorously evaluate the significance of the results. Additionally, all the surgical margins and specimens evaluated intraoperatively were negative for carcinoma or presented an outer rim of healthy tissue, thus impeding validation of the classifier intraoperatively for positive margin detection. To account for the lack of positive margins, we evaluated a test set of 12 prospectively collected clinical touch imprints that included IDC samples. While the accuracy obtained with touch imprints was moderate, the results are promising considering the low cellularity and tumor density of the touch imprints compared with the tissue samples from which the classifiers were developed, as well as the small sample size and the different nature of the samples (eg, cells on a glass slide) and of the experimental environment (eg, different mass spectrometer and laboratory settings). Overall, the results from touch imprint data indicate that the classifier is applicable for cancer detection across different settings, users, and sample types, although further validation intraoperatively is still necessary in tissues with positive margins.

Lastly, technical limitations concerning MS instrumentation and sampling area of the device are noted. Regarding instrumentation, no MS signal was detected for 27 of the 174 total analyses (16%) of breast tissue samples in the OR, attributable to instrument contamination and variabilities within the intraoperative setting, as previously described.^29^ Regarding sampling area, the pen tip area (5.73 mm^2^) provided by the devices used in this study was limited compared with the area of the in vivo surgical bed and/or the excised breast specimens, suggesting multiple analyses may be needed depending on margin dimensions and/or tissue coverage needs. Device designs with a large pen-tip diameter are possible and would enable increased area coverage, reduce the number of analyses needed, and potentially aid in quicker identification of cancerous regions in large specimens compared with smaller-diameter pen tips. Further studies using larger pen-tip diameters are needed to evaluate usability and clinical performance for assessment of large surgical specimens and potential limitations related to the sensitivity in detecting infiltrating tumors with low tumor cellularity.

## Conclusions

Results of this diagnostic study suggest that the MasSpec Pen technology can be used for rapid tissue assessment, with the system exhibiting robust clinical performance for discrimination of IDC from healthy breast tissue using banked tissue samples and showing potential for intraoperative use. This work suggested that this technology has potential to provide near real-time feedback to surgeons assessing surgical margins in vivo and on excised specimens. With further refinement and clinical testing, this platform may help reduce secondary BCS operations and economic costs,^38^ thus improving patient care and outcomes.

## Notes

Supplement 1. **eMethods 1.** MasSpec Pen Analysis of Banked Breast Tissue Samples **eMethods 2.** Intraoperative Clinical Testing of the MasSpec Pen During Breast Cancer Operations **eMethods 3.** Statistical Analysis **eFigure 1.** TIC Normalized Ion Abundances of Tentatively Identified Metabolite and Lipid Species in Banked Normal Breast and IDC Tissues **eFigure 2.** Representative Molecular Profile Obtained From In Vivo Analysis of a Sentinel Lymph Node **eFigure 3.** Average Molecular Profiles Obtained From Touch Imprints of Benign Tissue and Palpable IDC Tumor **eFigure 4.** Comparison of Representative Molecular Profiles Obtained From In Vivo Analyses of Normal Breast Tissue Collected at 2 Different Clinical Sites **eTable 1.** Summary of Lasso Prediction Results for Discriminating IDC From Normal Breast Using Banked Tissues **eTable 2.** Per-Case Prediction Results on Data Collected Intraoperatively With the MasSpec Pen Compared With Final Postoperative Pathology Notes **eTable 3.** Confusion Matrix of Touch Imprint Prediction Results **eTable 4.** Summary of Prediction Results Compared With Pathology Notes per Touch Imprint

Supplement 2.
Data Sharing Statement
