# Supplementary material for: Intraoperative Evaluation of Breast Tissues During Breast Cancer Operations Using the MasSpec Pen
Source: JAMA Netw Open. 2024 Mar 22;7(3):e242684. doi: 10.1001/jamanetworkopen.2024.2684 (PMC10960202; doi:10.1001/jamanetworkopen.2024.2684)
Supplement: Supplement 1. — eMethods 1. MasSpec Pen Analysis of Banked Breast Tissue Samples eMethods 2. Intraoperative Clinical Testing of the MasSpec Pen During Breast Cancer Operations eMethods 3. Statistical Analysis eFigure 1. TIC Normalized Ion Abundances of Tentatively Identified Metabolite and Lipid Species in Banked Normal Breast and IDC Tissues eFigure 2. Representative Molecular Profile Obtained From In Vivo Analysis of a Sentinel Lymph Node eFigure 3. Average Molecular Profiles Obtained From Touch Imprints of Benign Tissue and Palpable IDC Tumor eFigure 4. Comparison of Representative Molecular Profiles Obtained From In Vivo Analyses of Normal Breast Tissue Collected at 2 Different Clinical Sites eTable 1. Summary of Lasso Prediction Results for Discriminating IDC From Normal Breast Using Banked Tissues eTable 2. Per-Case Prediction Results on Data Collected Intraoperatively With the MasSpec Pen Compared With Final Postoperative Pathology Notes eTable 3. Confusion Matrix of Touch Imprint Prediction Results eTable 4. Summary of Prediction Results Compared With Pathology Notes per Touch Imprint [file jamanetwopen-e242684-s001.pdf]

Supplementary Online Content

Garza KY, King ME, Nagi C, et al. .Intraoperative evaluation of breast tissues during breast cancer operations using the MasSpec Pen *JAMA Netw Open*. 2024;7(3):e242684. doi:10.1001/jamanetworkopen.2024.2684

eMethods 1. Analysis of Banked Breast Tissue Samples
eMethods 2. Intraoperative Clinical Testing of the MasSpec Pen During Breast Cancer Operations
eMethods 3. Statistical Analysis
eFigure 1. TIC Normalized Ion Abundances of Tentatively Identified Metabolite and Lipid Species in Banked Normal Breast and IDC Tissues
eFigure 2. Representative Molecular Profile Obtained From In Vivo Analysis of a Sentinel Lymph Node
eFigure 3. Average Molecular Profiles Obtained From Touch Imprints of Benign Tissue and Palpable IDC Tumor
eFigure 4. Comparison of Representative Molecular Profiles Obtained From In Vivo Analyses of Normal Breast Tissue Collected at 2 Different Clinical Sites
eTable 1. Summary of Lasso Prediction Results for Discriminating IDC From Normal Breast Using Banked Tissues
eTable 2. Per-Case Prediction Results on Data Collected Intraoperatively Compared With Final Postoperative Pathology Notes
eTable 3. Confusion Matrix of Touch Imprint Prediction Results
eTable 4. Summary of Prediction Results Compared With Pathology Notes per Touch Imprint

This supplementary material has been provided by the authors to give readers additional information about their work.

© 2024 Garza KY et al. *JAMA Network Open*.

eMethods 1

Analysis of banked breast tissue samples

The MSPen coupled to a Q Exactive mass spectrometer (Thermo Fisher Scientific) was used to analyze the normal breast and IDC tissue samples in four batches in a research laboratory at The University of Texas at Austin (UT Austin). Tissues were thawed in ambient conditions prior to analysis. Data were collected in the negative ion mode from *m/z* 120-1800 using a resolving power of 140,000. For laboratory experiments, a tubing length of 1.5 m and a pen tip reservoir diameter of 2.7 mm (area 5.73 mm^2) was used.

eMethods 2

Intraoperative clinical testing of the MSPen during breast cancer operations

Patients were eligible for enrollment in our study if they were ≥ 18 years old and scheduled to undergo a lumpectomy, excisional biopsy, or mastectomy for IDC, ductal carcinoma in situ (DCIS), or other non-malignant conditions (e.g., fibroadenoma, atypical hyperplasia) irrespective of our study. Patients who agreed to participate in the study provided written consent.

eMethods 3

Statistical analysis

Histological features were carefully considered when extracting data to build classification models for disease prediction. Non-tissue specific peaks, which were identified by performing blank analyses of LC-MS grade water, were removed, and the data were normalized to the total ion count. Logistic regression regularized using the least absolute shrinkage and selection operator (lasso) was implemented via the glmnet package in the R CRAN language library to develop a classification model using only banked tissue samples. Training set performance was evaluated using leave-one-out cross validation (LOO-CV) and further assessed using a validation and independent test sets of samples (**Figure 1**). The validation set comprises data collected at the same time as the training set data; these data were randomly split to establish the training and validation sets. The test set of data refers to a completely independent data set used to

© 2024 Garza KY et al. *JAMA Network Open*.

evaluate the model's performance. Accuracy, sensitivity, and specificity for all prediction results were calculated based on agreement with pathological evaluation of the same tissues.

Analyses from three OR cases were excluded from statistical analysis due to invasive lobular carcinoma (ILC) post-surgical diagnosis (n=2 patients, 44 analyses total) or no signal obtained (n=1 patient, 9 analyses). Data were also excluded if they were from tissues not relevant to classification (e.g., lymph node) (n=46 analyses) or if no detectable signal was obtained (n=27 analyses). Thus, 147 remaining analyses from 22 cases were retained for statistical prediction. Lasso prediction results for *in vivo* tissues analyzed that were not resected, e.g. normal/uninvolved tissues, were compared to the gross assessment performed by the surgeon. Prediction results for *in vivo* and *ex vivo* data of the excised specimens were compared to postoperative final pathology reports. For touch imprint data, data resulting in no signal (n=5) or containing contamination from previous analyses (n=1) were excluded, thus the 12 remaining analyses from 10 patients were used for classification, using pathologic assessment of the stained slides for evaluation.

All of the statistical analysis were first performed by M.E.K. and independently repeated and verified by M.W.S. and R.T.

© 2024 Garza KY et al. *JAMA Network Open*.

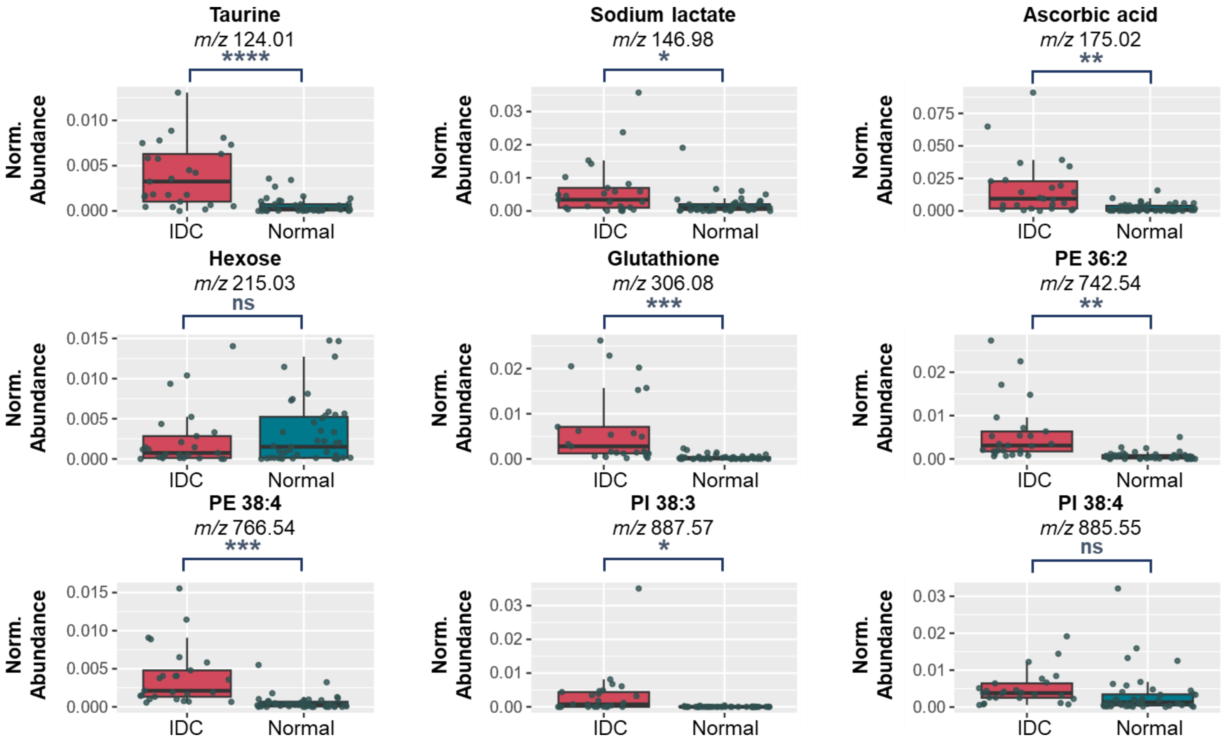

eFigure 1. TIC normalized ion abundances of tentatively identified metabolite and lipid species in banked normal breast (n=25) and IDC (n=43) tissues. ns = not significant, ($p > 0.05$); *$p < 0.05$; **$p < 0.01$, ***$p < 0.001$, ****$p < 0.0001$ (Student t-test with Benjamini-Hochberg correction).

© 2024 Garza KY et al. *JAMA Network Open*.

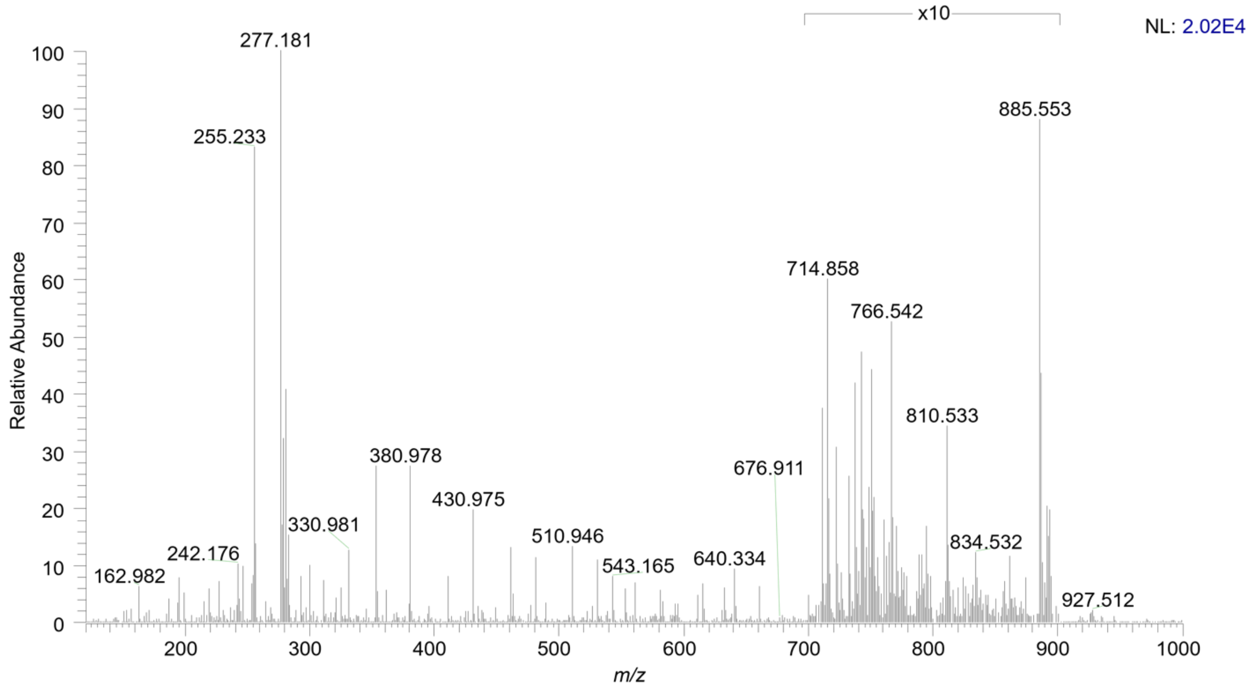

eFigure 2. Representative molecular profile obtained from in vivo analysis of a sentinel lymph node from patient Br0011.

© 2024 Garza KY et al. *JAMA Network Open*.

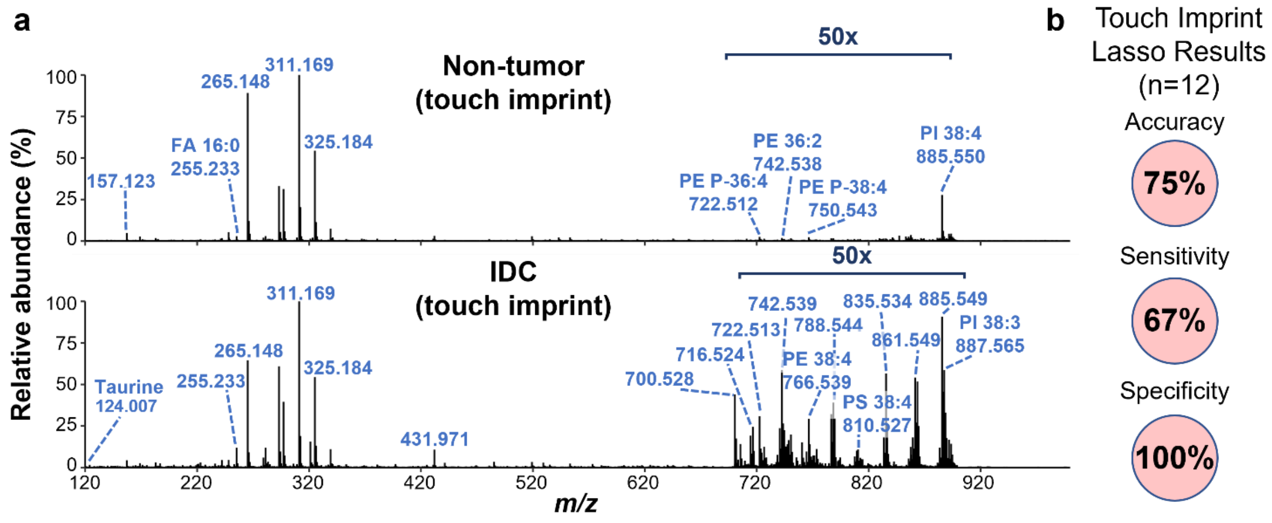

eFigure 3. **Average molecular profiles obtained from touch imprints of benign tissue (n=3 analyses) and palpable IDC tumor (n=9 analyses).**

© 2024 Garza KY et al. *JAMA Network Open*.

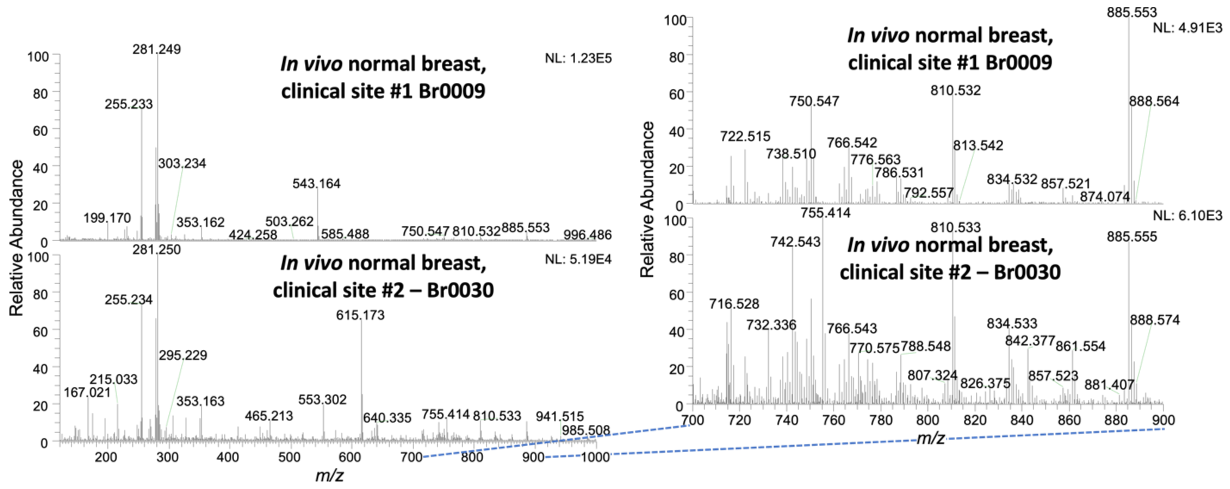

eFigure 4. Comparison of representative molecular profiles obtained from in vivo analyses of normal breast tissue collected at two different clinical sites. Mass spectra were collected from patient Br0009 (first clinical site) and Br0030 (second clinical site). Left panel shows full mass range. Right panel shows lipid region (*m/z* 700-900).

© 2024 Garza KY et al. *JAMA Network Open*.

eTable 1. Summary of lasso prediction results for discriminating IDC from normal breast using banked tissues.

**Data set**			**Lasso Prediction**		**Accuracy**	**Sensitivity**	**Specificity**
			Normal	Cancer			
Training Set	**Pathology Diagnosis**	Normal	**40**	3	95.6%	100.0%	93.0%
		Cancer	0	**25**			
Validation Set		Normal	**13**	1	95.5%	100.0%	92.9%
		Cancer	0	**8**			
Independent Test Set		Normal	**22**	0	90.6%	83.9%	100.0%
		Cancer	5	**26**			

© 2024 Garza KY et al. *JAMA Network Open*.

eTable 2. Per-case prediction results on data collected intraoperatively compared with final postoperative pathology notes.

Patient ID	Gross region analyzed[a]	MSP analysis	Lasso results	Postoperative pathology[b]	Agreement[c]
Br0001	Normal breast	IV	Normal	No residual carcinoma seen	Y
	Tumor	IV	Normal		Y
	Normal breast	EV	Normal		Y
	Normal breast	EV	Normal		Y
	Tumor	EV	Normal		Y
	Tumor	EV	Normal		Y
	Tumor	EV	Normal		Y
	Tumor	EV	Normal		Y
	Tumor	EV	Normal		Y
Br0002	Normal breast	EV	Normal	NA	Y
	Normal breast	EV	Normal		Y
Br0003	Normal breast	IV	Tumor	All margins negative for carcinoma	N
	Normal breast	IV	Normal		Y
	Normal breast	EV	Normal		Y
	Normal breast	EV	Normal		Y
Br0004	Surgical margin	IV	Normal	All margins negative for carcinoma	Y
	Surgical margin	IV	Normal		Y
	Normal breast	EV	Normal		Y
	Normal breast	EV	Normal		Y
	Normal breast	EV	Normal		Y
	Surgical margin	EV	Normal		Y
	Surgical margin	EV	Tumor		N
Br0005	Normal breast (left)	IV	Normal	All margins negative for carcinoma	Y
	Normal breast (left)	EV	Normal		Y
	Normal breast (left)	EV	Normal		Y
	Treated breast (right)	EV	Normal		Y
Br0007	Inferior, tumor adjacent margin	IV	Normal	All margins negative for carcinoma	Y
	Medial margin	IV	Normal		Y
	Tumor adjacent margin	EV	Normal		Y

© 2024 Garza KY et al. *JAMA Network Open*.

Patient ID	Gross region analyzed[a]	MSP analysis	Lasso results	Postoperative pathology[b]	Agreement[c]
	Tumor adjacent margin	EV	Normal		Y
Br0008	Normal breast (left)	IV	Normal	No residual carcinoma seen	Y
	Normal breast (left)	IV	Normal		Y
	Normal breast (right)	IV	Normal		Y
	Normal breast (right)	IV	Normal		Y
	Normal breast (right)	IV	Normal		Y
Br0009	Normal breast	IV	Normal	All margins negative for carcinoma	Y
	Normal breast	IV	Normal		Y
	Tumor	IV	Normal		Y
	Tumor	IV	Normal		Y
	Tumor	EV	Normal		Y
	Tumor	EV	Normal		Y
	Tumor	EV	Normal		Y
Br0010	Normal breast	IV	Normal	All margins negative for carcinoma	Y
	Normal breast	IV	Normal		Y
	Normal breast	IV	Normal		Y
	Tissue encapsulating tumor	IV	Normal		Y
	Tissue encapsulating tumor	IV	Normal		Y
	Tissue encapsulating tumor	IV	Normal		Y
	Super-lateral margin	EV	Normal		Y
	Super-lateral margin	EV	Normal		Y
Br0011	Tissue encapsulating tumor (left)	IV	Normal	All margins negative for carcinoma	Y
	Tissue encapsulating tumor (left)	IV	Normal		Y
	Tissue encapsulating tumor (left)	IV	Normal		Y
	Normal breast (right)	EV	Normal		Y
	Normal breast (right)	EV	Normal		Y
	Normal breast (right)	IV	Normal		Y
	Normal breast (right)	IV	Normal		Y
	Normal breast (right)	IV	Normal		Y
	Normal breast (left)	EV	Normal		Y
	Normal breast (left)	EV	Normal		Y

© 2024 Garza KY et al. *JAMA Network Open*.

Patient ID	Gross region analyzed[a]	MSP analysis	Lasso results	Postoperative pathology[b]	Agreement[c]
	Normal breast (left)	EV	Normal		Y
	Tissue encapsulating tumor (left)	EV	Normal		Y
	Tissue encapsulating tumor (left)	EV	Normal		Y
	Tissue encapsulating tumor (left)	EV	Normal		Y
	Tissue encapsulating tumor (left)	EV	Normal		Y
Br0012	Normal breast	IV	Normal	All margins negative for carcinoma	Y
	Tumor margin	IV	Normal		Y
	Tumor margin	IV	Normal		Y
	Tumor margin	IV	Normal		Y
	Tumor margin	IV	Normal		Y
Br0013	Superior anterior margin	IV	Normal	All margins negative for carcinoma	Y
	Superior anterior margin	IV	Normal		Y
	Superior margin	IV	Normal		Y
	Superior margin	IV	Normal		Y
	Deep margin, posterior	IV	Normal		Y
	Deep margin	IV	Normal		Y
	Deep margin	IV	Normal		Y
	Deep margin	IV	Normal		Y
	Superior anterior margin	EV	Normal		Y
	Superior anterior margin	EV	Normal		Y
Br0014	Anterior-lateral margin	EV	Normal	All margins negative for carcinoma	Y
	Anterior-lateral margin	EV	Normal		Y
	Inferior-superior margin	EV	Tumor		N
	Inferior-superior margin	EV	Normal		Y
Br0015	Normal breast	IV	Normal	All margins negative for carcinoma	Y
	Normal breast	IV	Normal		Y
	Normal breast	IV	Normal		Y
	Posterior margin	EV	Normal		Y
	Anterior-lateral margin	EV	Normal		Y
	Anterior-lateral margin	EV	Normal		Y
	Anterior-lateral margin	EV	Normal		Y

© 2024 Garza KY et al. *JAMA Network Open*.

Patient ID	Gross region analyzed[a]	MSP analysis	Lasso results	Postoperative pathology[b]	Agreement[c]
	Superior margin	EV	Normal		Y
	Superior margin	EV	Normal		Y
	Superior margin	EV	Normal		Y
Br0018	Normal breast (left)	IV	Normal	No residual carcinoma seen	Y
	Normal breast (left)	IV	Normal		Y
	Normal breast (left)	IV	Normal		Y
	Normal breast (left)	IV	Normal		Y
	Treated breast (right)	IV	Normal		Y
	Treated breast (right)	IV	Normal		Y
	Superior margin (left)	EV	Normal		Y
	Superior margin (left)	EV	Normal		Y
	Superior margin (right)	EV	Normal		Y
	Superior margin (right)	EV	Normal		Y
	Superior margin (right)	EV	Normal		Y
Br0019	Inferior margin	EV	Normal	All margins negative for carcinoma	Y
	Inferior margin	EV	Normal		Y
	Inferior margin	EV	Normal		Y
	Inferior margin	EV	Normal		Y
	Inferior margin	EV	Normal		Y
	Inferior margin	EV	Normal		Y
	Deep margin	EV	Normal		Y
	Deep margin	EV	Normal		Y
Br0020	Normal breast (left)	EV	Normal	Negative for carcinoma	Y
	Normal breast (left)	EV	Normal		Y
	Normal breast (right)	IV	Normal		Y
Br0023	Normal breast	IV	Normal	All margins negative for carcinoma	Y
	Normal breast	IV	Normal		Y
	Tissue encapsulating tumor	IV	Normal		Y
	Tissue encapsulating tumor	IV	Normal		Y
	Tissue encapsulating tumor	IV	Normal		Y
	Tissue encapsulating tumor	EV	Tumor		N

© 2024 Garza KY et al. *JAMA Network Open*.

Patient ID	Gross region analyzed[a]	MSP analysis	Lasso results	Postoperative pathology[b]	Agreement[c]
	Tissue encapsulating tumor	EV	Tumor		N
	Tissue encapsulating tumor	EV	Tumor		N
Br0026	Superior-lateral margin	IV	Normal	Specimen negative for carcinoma (benign fibroadenoma)	Y
	Superior-lateral margin	IV	Normal		Y
	Fibroadenoma	EV	Normal		Y
	Fibroadenoma	EV	Normal		Y
	Fibroadenoma	EV	Normal		Y
Br0030	Normal breast	IV	Normal	All margins negative for carcinoma	Y
	Normal breast	IV	Normal		Y
	Inferior margin (re-excised)	IV	Normal		Y
	Inferior margin (re-excised)	IV	Normal		Y
	Lateral margin	EV	Normal		Y
	Lateral margin	EV	Normal		Y
	Inferior margin (re-excised)	EV	Normal		Y
	Inferior margin (re-excised)	EV	Normal		Y
	Posterior margin	EV	Normal		Y
	Posterior margin	EV	Normal		Y
Br0031	Median margin	EV	Normal	All margins negative for carcinoma	Y
	Median margin	EV	Normal		Y
	Superficial margin	EV	Normal		Y
	Superficial margin	EV	Normal		Y
Br0032	Posterior margin	IV	Normal	All margins negative for carcinoma	Y
	Posterior margin	IV	Normal		Y
	Posterior aspect of tumor	EV	Normal		Y
	Posterior aspect of tumor	EV	Normal		Y

a. Based on intraoperative evaluation of tissue anatomy by surgeon.
b. Note that pathology reports only apply to analyses grossly identified as tumor and margin regions.
c. Based on agreement between lasso predictions and postoperative pathology notes.

Abbreviations: EV, ex vivo; ID, identification; IV, in vivo; MSP, MasSpec Pen; NA, not applicable.

© 2024 Garza KY et al. *JAMA Network Open*.

eTable 3. Confusion matrix of touch imprint prediction results.

		Lasso Prediction		Accuracy	Sensitivity	Specificity
		Normal	Cancer			
**Pathology Diagnosis**	Normal	**3**	0	75%	67%	100%
	Cancer	3	**6**			

© 2024 Garza KY et al. *JAMA Network Open*.

eTable 4. Summary of prediction results compared with pathology notes per touch imprint.

Sample	Lasso Prediction	Pathology notes	Agreement
Br0036_slide1_2	Cancer	All tumor cells	✓
Br0036_slide1_3	Cancer	All tumor cells	✓
Br0042	Normal	Fat, some macrophages in region of analysis	✓
Br0045_2	Normal	No tumor cells remaining in tumor bed, potential necrosis	✓
Br0051_1	Normal	All tumor cells	✗
Br0054	Cancer	All tumor cells	✓
Br0059_2	Normal	<5% tumor cells, with some more concentrated tumor cells outside of region of analysis	✗
Br0059_3	Cancer	10-15% tumor cells	✓
Br0063_1	Cancer	All tumor cells	✓
Br0065_2	Normal	Difficult to diagnose, not much cellular detail likely due to neoadjuvant treatment response; outside of region of analysis there are some clusters of disparate tumor cells	✓
Br0067_2	Cancer	All tumor cells	✓
Br0075_2	Normal	<5% tumor cells, not much other cellular material	✗

© 2024 Garza KY et al. *JAMA Network Open*.
