# Supplementary material for: Intraoperative Evaluation of Breast Tissues During Breast Cancer Operations Using the MasSpec Pen
Source: JAMA Netw Open. 2024 Mar 22;7(3):e242684. doi: 10.1001/jamanetworkopen.2024.2684 (PMC10960202; doi:10.1001/jamanetworkopen.2024.2684)
Supplement: Supplement 2. — Data Sharing Statement [file jamanetwopen-e242684-s002.pdf]

Data Sharing Statement

Garza. Intraoperative Evaluation of Breast Tissues During Breast Cancer Operations Using the MasSpec Pen. *JAMA Netw Open*. Published March 18, 2024.
doi:10.1001/jamanetworkopen.2024.2684

Data
Data available: Yes
Data types: Deidentified participant data
How to access data: data will be available through our laboratory repository in https://dataverse.org/
When available: With publication

Supporting Documents
Document types: None

Additional Information
Who can access the data: researchers
Types of analyses: statistical analysis
Mechanisms of data availability: with investigator support
